# Impact of factors on treatment outcomes in cervical degenerative disc disease: a logistic regression analysis of anterior decompression and interbody fusion with BAK/C technique

**DOI:** 10.3389/fsurg.2025.1637376

**Published:** 2025-09-24

**Authors:** Liang Hao, Aobo Zhang, Fengming Zhao, Honglei Liu, Xiaoli Sun

**Affiliations:** ^1^ Department of Neurosurgery, The Third Hospital of Shijiazhuang, Shijiazhuang, China; ^2^ Department of Neurosurgery, The Second Hospital of Hebei Medical University, Shijiazhuang, China

**Keywords:** anterior cervical discectomy and fusion, BAK/C interbody fusion, cervical disc degenerative disease, pseudoarthrosis, bone mineral density, logistic regression analysis

## Abstract

**Objectives:**

To identify predictive prognostic factors through logistic regression analysis in patients with cervical degenerative disc disease (CDDD) undergoing anterior cervical discectomy and fusion (ACDF) combined with the Bagby and Kuslich (BAK/C) interbody fusion technique.

**Methods:**

This retrospective study included 80 patients treated with ACDF and BAK/C between January and December 2020, with a 3-year follow-up. Patients were stratified into a control group (favorable recovery, *n* = 52) and an observation group (poor recovery, *n* = 28) based on pain relief and neurological improvement. Radiological fusion rates and Japanese Orthopaedic Association (JOA) scores were evaluated. Multivariate logistic regression was performed to assess independent predictors of outcomes.

**Results:**

The control group exhibited significant JOA score improvement at the final follow-up (14.49 ± 0.25 vs. preoperative 10.74 ± 1.16, *P* < 0.001), while the observation group showed limited recovery (12.19 ± 0.32 vs. preoperative 11.15 ± 1.45, *P* < 0.001). The overall fusion rate was significantly higher in the control group (92.3% vs. 64.3%, *P* = 0.002). Multivariate analysis identified age ≥55 years (observation group: 62.35 ± 5.41 vs. control: 51.47 ± 6.37, *P* < 0.001), reduced bone mineral density (T-score: −2.1 ± 0.8 vs. −1.3 ± 0.6, *P* < 0.001), postoperative complications (46.4% vs. 13.5%, *P* = 0.003), and baseline disease severity as independent risk factors for poor outcomes (*P* < 0.05). The observation group demonstrated significantly higher pseudoarthrosis rates (35.7% vs. 9.6%, *P* = 0.003).

**Conclusion:**

Advanced age, low bone density, and postoperative complications critically compromise outcomes of ACDF with BAK/C fusion. Preoperative bone density optimization, judicious use of augmented multi-level fixation, and precision patient selection are pivotal for improving prognosis. These findings provide evidence-based insights for individualized clinical decision-making.

## Introduction

Degenerative diseases of the cervical intervertebral disc involve pathophysiological processes such as the reduction of intervertebral disc height and spinal cord compression due to disc degeneration, commonly referred to as cervical spondylosis (1). With an aging population and changing lifestyles, the prevalence of cervical degenerative disc disease (CDDD) is steadily increasing (2, 3). This condition significantly affects patients' quality of life, often leading to symptoms such as neck and shoulder pain, upper limb radiation pain, and sensory and motor dysfunction. These symptoms can severely impair patients' ability to work and engage in normal daily activities.

Anterior cervical discectomy and fusion (ACDF) is a widely used and effective surgical approach for treating CDDD. ACDF involves removing the intervertebral disc and bone tissue compressing the nerve structures through an anterior cervical incision, thereby alleviating nerve compression symptoms (4). Bone grafts and interbody devices are typically implanted to maintain intervertebral space stability and promote bone fusion (5). The main advantage of ACDF is the direct removal of the compressed structures, such as intervertebral discs and osteophytes, which relieves pressure on the nerves. Additionally, modern ACDF techniques are associated with high success rates, minimal trauma, and relatively quick postoperative recovery, with success rates exceeding 90% reported in contemporary practices (6). ACDF is effective for both single and multiple levels of degenerative disc disease, particularly in patients with prominent nerve root compression symptoms (7).

The Brantigan, Allograft, and Kuslich/Cadaveric (BAK/C) interbody fusion technique, a classic threaded cage design, is commonly used in conjunction with ACDF surgery to enhance the fusion success rate and stability (8). Despite increasing adoption of newer cage designs made from materials like polyetheretherketone (PEEK) or porous titanium, the BAK/C technique remains clinically relevant, particularly in resource-limited settings, due to its cost-effectiveness and proven mid-term outcomes (9). During BAK/C fusion, bone grafts—often sourced from the patient's own body or synthetic materials—are placed in the intervertebral space to promote vertebral fusion, thereby increasing the stability of the intervertebral space (10). This technique helps reduce postoperative intervertebral motion and alleviates cervical pain in patients. The BAK/C fusion method is particularly beneficial for patients with severe degenerative disc disease, unstable intervertebral spaces, or extensive disc resections.

However, despite the widespread clinical use of ACDF and BAK/C techniques, patient responses to treatment can vary significantly. While some patients experience substantial improvements in neurological function and pain relief, others may show minimal improvements or even worsening symptoms (11, 12). We hypothesize that patient-specific factors (age, bone quality, and postoperative care) rather than technical variables primarily determine clinical outcomes in BAK/C-assisted ACDF. Consequently, it is essential to explore the factors influencing the effectiveness of ACDF combined with BAK/C interbody fusion for treating CDDD. Previous studies have focused predominantly on implant-related factors (13), with limited analysis of modifiable patient characteristics. Understanding these predictors can help clinicians optimize treatment plans and improve surgical outcomes for patients (14).

This study retrospectively analyzed 80 patients with CDDD treated at our hospital between January and December 2020. The primary focus was to explore the factors influencing the curative effect of ACDF combined with BAK/C interbody fusion, through logistic regression analysis. This comprehensive factor analysis addresses a critical gap in existing literature by evaluating both surgical and patient-related variables over a 3-year follow-up period.

## Methods and materials

### Ethical considerations

This study received approval from The Third Hospital of Shijiazhuang Ethics Committee. The study was conducted in accordance with the Declaration of Helsinki. All participants provided written informed consent for retrospective data analysis through opt-out methodology, with personal identifiers removed during data processing.

### Patient enrollment process

**Figure 1:**
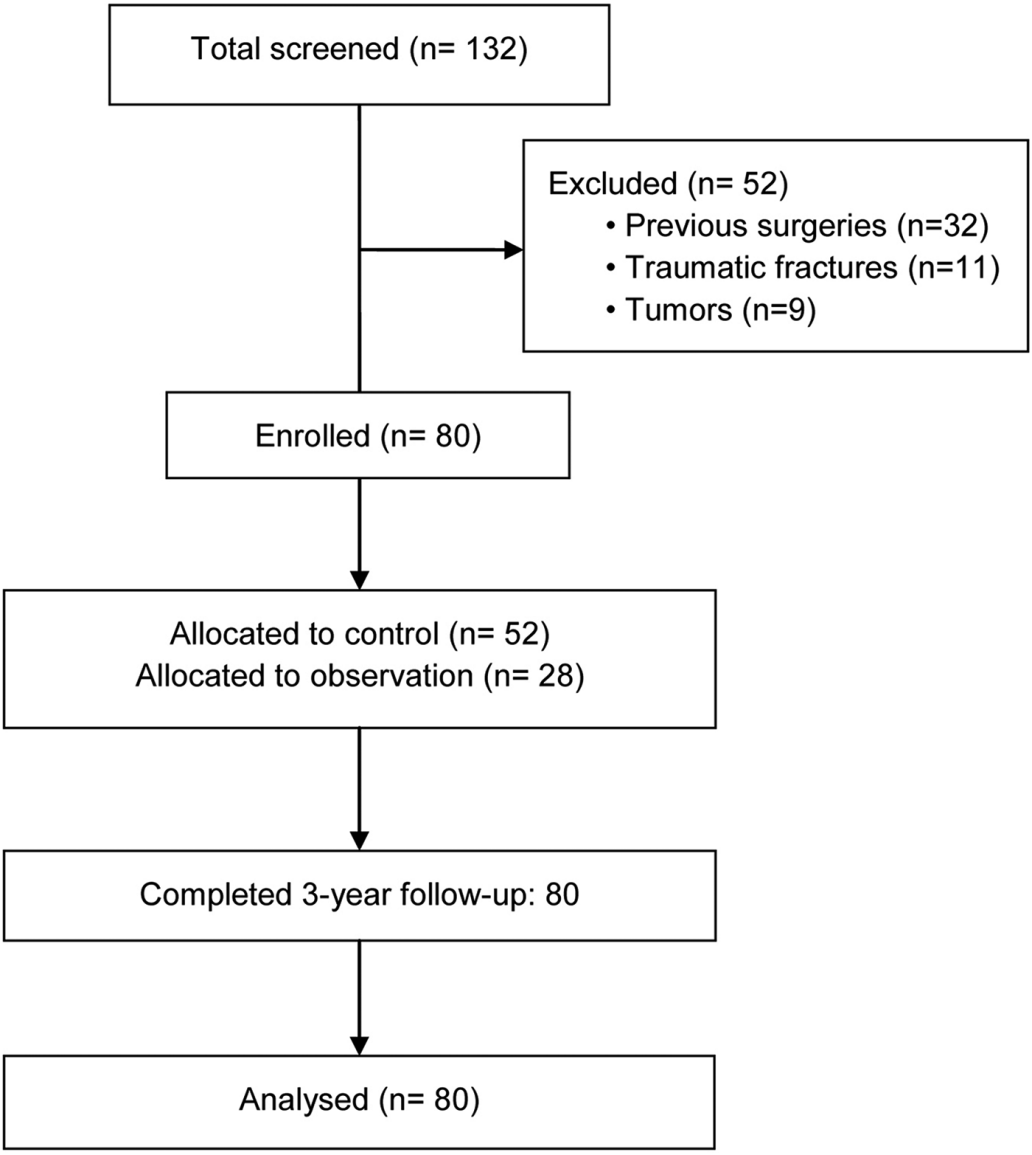
The study flow diagram.

Between January-December 2020, 132 consecutive patients with CDDD were screened for eligibility at our institution. After excluding 52 patients (32 with previous cervical surgeries, 11 with traumatic fractures, and 9 with tumors), 80 patients met the inclusion criteria. These patients were subsequently divided into a control group (*n* = 52) and an observation group (*n* = 28) based on their 3-year follow-up outcomes (Figure 1).

### General information

A retrospective analysis was conducted on 80 hospitalized patients with degenerative cervical intervertebral disc disease at our hospital from January to December 2020. All patients underwent ACDF surgery using the BAK/C technique. The age of the patients ranged from 36–75 years (mean 55.36 ± 7.28). Of the patients, 57 were male and 23 were female. All patients had a complete 3-year follow-up. Based on clinical outcomes reported at follow-up, the patients were divided into two groups: the control group (good neurological recovery, *n* = 52) and the observation group (minimal or worsened neurological recovery, *n* = 28). Good recovery was defined as having a postoperative Visual Analog Scale (VAS) score for neck/arm pain of ≤2 (representing minimal to no residual pain) combined with a Japanese Orthopaedic Association (JOA) score improvement of ≥4 points, which is considered a significant neurological recovery (15). Poor recovery was defined as a VAS score >2 with a JOA improvement of <2 points or any neurological deterioration (15). These thresholds were based on clinically meaningful changes, where a VAS score ≤2 correlates with excellent pain control (16), and a JOA improvement ≥4 points reflects substantial functional restoration (15). The combination of these was used to stringently define a favorable outcome. While other methods like ROC curve analysis could derive statistical cutoffs, our approach used established clinical benchmarks to ensure the relevance of the outcome groups.

### Inclusion criteria

• Patients aged ≥ 18 years, regardless of gender.
• Diagnosed with degenerative cervical intervertebral disc disease based on clinical and imaging findings.
• Significant pain and/or neurological symptoms impacting quality of life, requiring surgery.
• Underwent anterior decompression and BAK/C interbody fusion.
• Availability of complete data for a minimum 3-year follow-up.

### Exclusion criteria

• Serious cervical conditions (e.g., fracture, infection, tumor).
• Previous cervical surgery.
• Pregnant or lactating women.

### Surgical technique

Under general anesthesia, patients were positioned supine with slight neck extension. A right anterior approach was used to expose the cervical spine. The anterior longitudinal ligament was opened, and the intervertebral space was distracted using a Casper distractor. After discectomy, a reamer was used to enlarge the disc space, and osteophytes were removed using a micro curette or Kerrison rongeur. All surgical procedures were performed by a team of two senior neurosurgeons (L.H. with >10 years experience, and another with >15 years experience) to minimize variability in technique. A standardized threaded cylindrical titanium alloy BAK/C cage (Zimmer Biomet, Warsaw, IN, USA) was consistently used in all patients. Anterior cervical plates were selectively used in multi-level procedures (*n* = 14/22 multi-level cases) based on intraoperative stability assessment using the White-Panjabi criteria (17). BAK/C fusion and autogenous bone fragments, harvested locally from the vertebral bodies, were used to repair spinal defects. Eight patients received additional bone grafting from the iliac crest due to poor local bone stock. Postoperatively, all patients wore a Philadelphia hard neck brace for 2 months.

### Radiological evaluation

**Figure 2:**
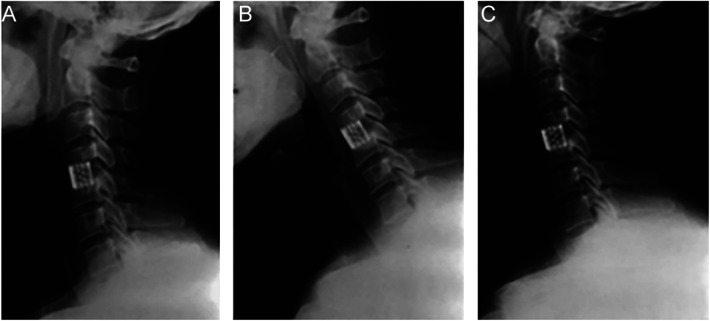
Representative radiographs of a patient undergoing single-level ACDF with a BAK/C cage at C5–6. **(A)** Preoperative lateral x-ray showing disc space narrowing and osteophyte formation. **(B)** Lateral x-ray at 1 week post-op showing cage placement and restored intervertebral height. **(C)** Lateral x-ray at the final 3-year follow-up demonstrating solid bony fusion and maintained alignment.

X-rays were obtained before surgery, 1 week post-op, at 6 months, and at the final follow-up. Bone mineral density was assessed preoperatively using dual-energy x-ray absorptiometry (DXA) at the lumbar spine (GE Lunar Prodigy). Bone density was reported using the T-score, which compares a patient's BMD to that of a healthy, young adult reference population. The D-value, defined as the sagittal diameter of the spinal canal at the most compressed level, was measured on preoperative T2-weighted sagittal MRI scans to quantify the degree of baseline stenosis. Anteroposterior, lateral, and dynamic flexion-extension x-rays were evaluated independently by two spinal surgeons and one radiologist blinded to clinical outcomes. Firm fusion was indicated when both of the following criteria were met: (1) segmental motion on extension-flexion radiographs <2 degrees, and (2) bridging trabecular bone across ≥50% of the graft-host interface on CT scans (18). While CT scans were not routinely obtained for all patients at final follow-up, they were mandated for all cases where non-union was suspected on dynamic flexion-extension radiographs. This protocol ensured CT confirmation for all 15 cases of pseudoarthrosis reported in this study. Figure 2 shows primary fusion with BAK/C. The intervertebral height (anterior and posterior edges) was measured, and changes were calculated between post-op and final follow-up.

### JOA score

The JOA scoring system (19) was used to assess neurological impairment in cervical spondylosis patients, evaluating sensory, motor, and activity functions. The total JOA score ranged from 0–17, with lower scores indicating more severe impairment. Neurological recovery rate was calculated as: (Postoperative score—Preoperative score)/(17—Preoperative score) × 100%. Scores of 15–17 were considered mild, 11–14 moderate, and 0–10 severe. The JOA score helped assess the neurological function and severity of the disease.

### Statistical analysis

A *post-hoc* sensitivity analysis was performed using G*Power software (version 3.1) to determine the minimum effect size our study could reliably detect (20). With our sample sizes (*n* = 52 in the control group, *n* = 28 in the observation group), an alpha of 0.05, and a power of 80%, the analysis indicated that our study was sufficiently powered to detect an odds ratio of 2.75 or greater for the primary binary predictors in the logistic regression model. This confirms the adequacy of our sample size for identifying clinically meaningful risk factors. Data are presented as mean ± standard deviation for continuous variables and as frequencies (percentages) for categorical variables. The Student's t-test or Mann–Whitney *U* test was used for continuous data, and the chi-square test or Fisher's exact test was used for categorical data to compare groups. Complications were classified using the Spinal Adverse Events Severity system (21), categorized as intraoperative, early postoperative (<30 days), or late postoperative (>30 days). A *P*-value < 0.05 was considered statistically significant. Statistical analysis was conducted using SPSS version 26.0 (SPSS Inc., Chicago, IL, USA).

## Results

### General data analysis

The baseline characteristics of the two groups are presented in Table 1. The male-to-female ratio in the control group was 37:15, with an average age of 51.47 ± 6.37 years and a BMI of 22.49 ± 1.88 kg/m². In the observation group, the male-to-female ratio was 20:8, with an average age of 62.35 ± 5.41 years and a BMI of 23.85 ± 1.23 kg/m². The average age of the observation group was significantly higher than that of the control group (*P* = 0.015). No significant differences were found in sex, BMI, hypertension, diabetes, smoking, or alcohol use between the two groups (*P* > 0.05).

**Table 1:** General data analysis.

Parameter	Control Group (*n* = 52)	Observation Group (*n* = 28)	T/*χ*² Value	*P*-value
Sex (Male:Female)	37:15	20:8	0.003	0.958
Age (years)	51.47 ± 6.37	62.35 ± 5.41	−8.157	**<0** **.** **001**
BMI (kg/m²)	22.49 ± 1.88	23.85 ± 1.23	−3.561	0.061
Hypertension	11 (21.2%)	8 (28.6%)	0.435	0.509
Diabetes	6 (11.5%)	3 (10.7%)	0.016	0.900
Smoking	18 (34.6%)	11 (39.3%)	0.177	0.674
Alcohol use	15 (28.8%)	8 (28.6%)	0.001	0.978

Bold values indicate a statistically significant difference (*P* < 0.05).

### Fusion segment distribution

The distribution and fusion levels of the segments are detailed in Table 2. In the control group, ACDF with BAK/C involved 66 segments, with C4–5 being the most common level. Definitive fusion was achieved in 61/66 segments (92.4%). In the observation group, the procedure involved 35 segments, with C5–6 being the most common level. Definitive fusion was achieved in only 22/35 segments (62.9%). There were no significant differences in the distribution of operated levels or the number of levels fused (one, two, or adjacent) between the two groups (*P* > 0.05).

**Table 2:** Distribution and fusion level of fusion segments.

Index	Control group (*n* = 52)	Observation group (*n* = 28)	T/χ² value	*P*-value
Fusion stage distribution (Total segments)				
C3–4	15/66 (22.7%)	8/35 (22.9%)	0.001	0.979
C4–5	24/66 (36.4%)	11/35 (31.4%)	0.292	0.589
C5–6	20/66 (30.3%)	12/35 (34.3%)	0.199	0.655
C6–7	7/66 (10.6%)	4/35 (11.4%)	0.022	0.883
Fusion level (per patient)				
One-level fusion	36 (69.2%)	19 (67.9%)	0.015	0.904
Two-level fusion	8 (15.4%)	4 (14.3%)	0.024	0.876
Adjacent level fusion	8 (15.4%)	5 (17.9%)	0.103	0.748

### JOA scores of patients treated with ACDF and BAK/C

**Figure 3:**
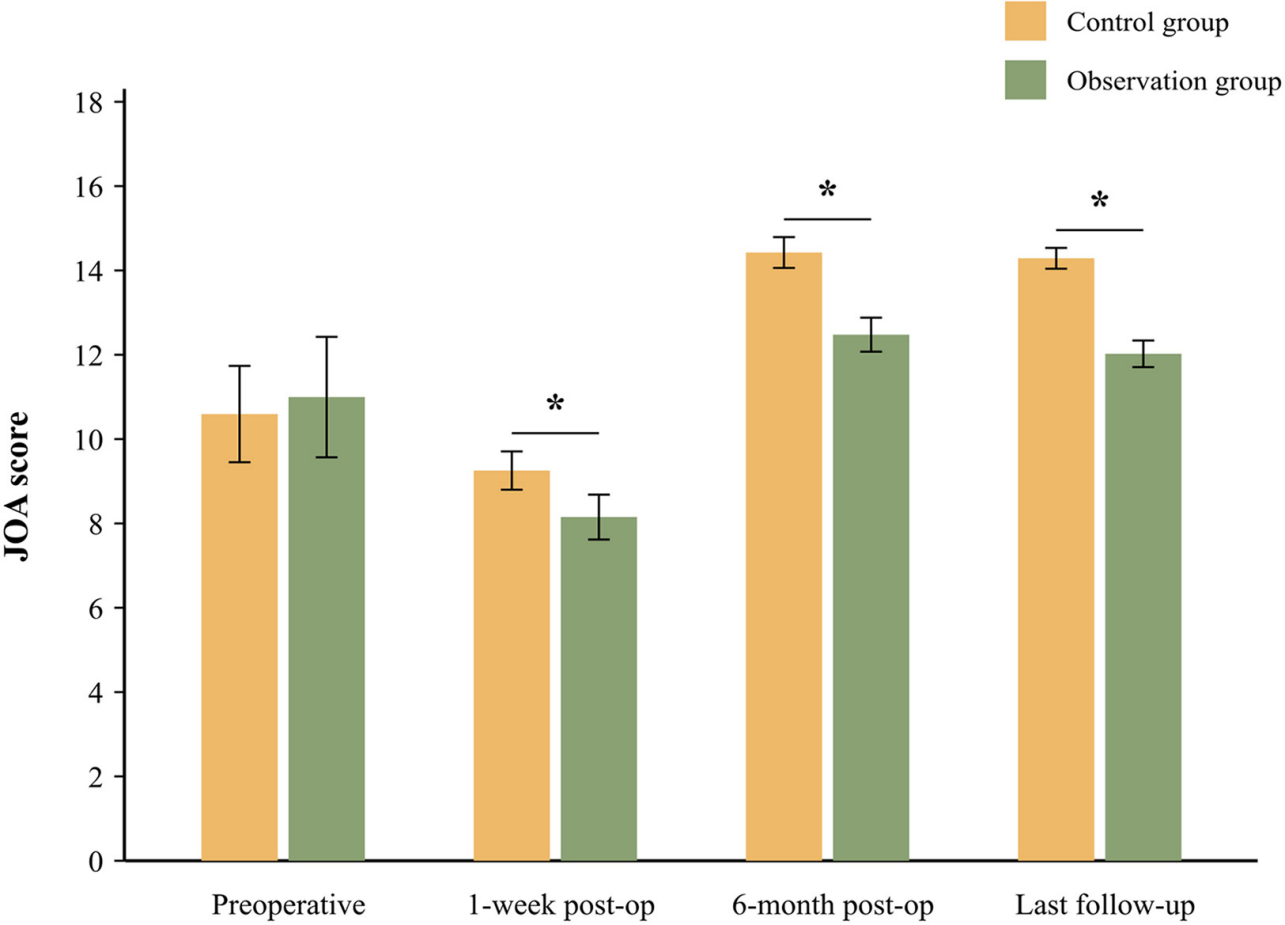
JOA scores of patients before and after the operation. Error bars represent standard deviation. *P < 0.05 indicates a significant difference between the control and observation groups at the specified time point.

The JOA scores were used to evaluate cervical nerve function over time (Figure 3, Table 3). Preoperative JOA scores were comparable between groups (control: 10.74 ± 1.16 vs. observation: 11.15 ± 1.45, *P* = 0.475). At one week post-surgery, both groups showed transient JOA score reductions (control: 9.38 ± 0.46 vs. observation: 8.26 ± 0.54), though the difference was not statistically significant (*P* = 0.021). By the 6-month follow-up, the control group demonstrated superior recovery (14.63 ± 0.37 vs. 12.65 ± 0.41, *P* = 0.035), with sustained significant differences at the final follow-up (14.49 ± 0.25 vs. 12.19 ± 0.32, *P* = 0.011).

**Table 3:** JOA scores of patients before and after the operation.

Group	Before operation	1-week post-op	6-month post-op	Last follow-up
Control group (*n* = 52)	10.74 ± 1.16	9.38 ± 0.46	14.63 ± 0.37	14.49 ± 0.25
Observation group (*n* = 28)	11.15 ± 1.45	8.26 ± 0.54	12.65 ± 0.41	12.19 ± 0.32
*T* value	−1.254	8.471	19.520	28.324
*P* value	0.214	**0.021**	**0.035**	**0.011**

Bold values indicate a statistically significant difference (*P* < 0.05).

### Radiological analysis of patients treated with ACDF and BAK/C

Radiological analysis revealed significant differences in key parameters (Table 4). The preoperative D-value (spinal canal diameter) was significantly lower in the observation group compared to the control group (3.85 ± 0.57 mm vs. 5.23 ± 0.63 mm, *P* = 0.041), indicating more severe baseline stenosis. Lumbar spine bone mineral density was also significantly lower in the observation group (T-score: −2.1 ± 0.8 vs. −1.3 ± 0.6, *P* < 0.001). The loss of intervertebral height at final follow-up was significantly greater in the observation group (−1.45 ± 0.21 mm vs. −0.79 ± 0.17 mm, *P* < 0.001). These findings indicate that the structural maintenance of the cervical vertebrae in the observation group was less effective compared to the control group.

**Table 4:** Radiographic parameters.

Parameter	Control group (*n* = 52)	Observation group (*n* = 28)	T value	*P* value
*D* value (mm)	5.23 ± 0.63	3.85 ± 0.57	2.071	**0** **.** **041**
Intervertebral height change (mm)	−0.79 ± 0.17	−1.45 ± 0.21	2.980	**0** **.** **004**
Bone Density (T-score)	−1.3 ± 0.6	−2.1 ± 0.8	5.112	**<0** **.** **001**

Bold values indicate a statistically significant difference (*P* < 0.05).

### Analysis of factors affecting treatment outcome

A univariate analysis was performed to examine factors influencing the treatment outcomes of ACDF combined with BAK/C fusion (Table 5). According to the preoperative JOA scores, the initial condition of patients in the observation group was more severe than in the control group. Significant differences were found between the two groups in terms of age, bone mineral density, rate of postoperative complications, initial condition, and patients' self-reported postoperative care adherence (*P* < 0.05).

**Table 5:** Analysis of related factors of treatment effect.

Index	Control group (*n* = 52)	Observation group (*n* = 28)	*P* value
Age			**0** **.** **005**
<55 years	34 (65.4%)	9 (32.1%)	
≥55 years	18 (34.6%)	19 (67.9%)	
Bone mineral density			**0** **.** **008**
T-score ≥ −1.5 (Normal/Osteopenia)	35 (67.3%)	9 (32.1%)	
T-score < −1.5 (Osteopenia/Osteoporosis)	17 (32.7%)	19 (67.9%)	
Postoperative complications			**0** **.** **003**
Presence	7 (13.5%)	13 (46.4%)	
Absence	45 (86.5%)	15 (53.6%)	
Preoperative JOA score			**0** **.** **036**
15–17 (Mild)	6 (11.5%)	1 (3.6%)	
11–14 (Moderate)	42 (80.8%)	18 (64.3%)	
0–10 (Severe)	4 (7.7%)	9 (32.1%)	
Postoperative care adherence			**0** **.** **002**
Strong	35 (67.3%)	13 (46.4%)	
Common	11 (21.2%)	5 (17.9%)	
Weak	6 (11.5%)	10 (35.7%)	

Bold values indicate a statistically significant difference (*P* < 0.05).

### Logistic regression analysis of factors influencing therapeutic effect

Multivariate logistic regression analysis revealed that age ≥55 years, low bone mineral density (T-score < −1.5), severe initial condition (preoperative JOA score ≤ 10), the presence of postoperative complications, and weak postoperative care adherence were all significant independent risk factors influencing the efficacy of ACDF combined with BAK/C interbody fusion in treating CDDD (*P* < 0.05) (Table 6).

**Table 6:** Logistic regression analysis of influencing factors of therapeutic effect of ACDF and BAK/C.

Variable	*β*	Odds Ratio (OR)	95% CI	*P* value
Age (≥55 vs. < 55)	1.35	3.86	1.25–11.89	**0** **.** **019**
Bone density (T-score < −1.5)	1.28	3.60	1.19–10.88	**0** **.** **023**
Initial condition (JOA ≤ 10)	1.49	4.44	1.30–15.15	**0** **.** **017**
Complications (Presence)	1.75	5.75	1.73–19.14	**0** **.** **004**
Postoperative care (Weak)	1.61	5.00	1.42–17.58	**0** **.** **012**

Bold values indicate a statistically significant difference (*P* < 0.05).

### Surgical outcomes

A dedicated analysis of surgical parameters (Table 7) revealed critical differences between groups. The control group demonstrated a significantly higher fusion rate in single-level procedures (94.4% vs. 78.9%, *P* = 0.03). Postoperative complications were significantly more frequent in the observation group, including persistent dysphagia (>6 weeks) and most notably, pseudoarthrosis (35.7% vs. 9.6%, *P* = 0.003). In multi-level cases, the use of anterior plating was significantly higher in the control group (100% vs. 42.9%, *P* = 0.001), correlating with better fusion outcomes in that cohort.

**Table 7:** Surgical outcomes comparison.

Parameter	Control Group (*n* = 52)	Observation Group (*n* = 28)	*P*-value
Fusion rate			
- Total segments	61/66 (92.4%)	22/35 (62.9%)	**0** **.** **002**
- Single-level patients	34/36 (94.4%)	15/19 (78.9%)	**0** **.** **030**
Complications			
- C5 palsy	2 (3.8%)	4 (14.3%)	0.124
- Dysphagia >6 weeks	3 (5.8%)	6 (21.4%)	**0** **.** **021**
- Pseudoarthrosis	5 (9.6%)	10 (35.7%)	**0** **.** **003**
Anterior plate usage (in multi-level cases only)	8/8 (100%)	6/14 (42.9%)	**0** **.** **013**

Bold values indicate a statistically significant difference (*P* < 0.05).

## Discussion

Degenerative disease of the cervical intervertebral disc is a common condition, with its prevalence increasing due to the aging population. Anterior cervical discectomy and fusion (ACDF) remains a gold standard for surgical treatment, but the effectiveness may vary among patients (22). Therefore, investigating the factors influencing the treatment outcomes of these surgeries is crucial to making precise, evidence-based clinical decisions, ultimately improving therapeutic outcomes and patients' quality of life.

In this study, we retrospectively analyzed 80 patients who underwent ACDF and BAK/C interbody fusion for the treatment of CDDD. The aim was to explore the influencing factors affecting treatment efficacy. Multivariate logistic regression analysis revealed that advanced age, low bone mineral density, severe initial condition, postoperative complications, and poor postoperative care adherence were the primary factors influencing the treatment outcomes.

We identified age as a crucial factor significantly affecting treatment outcomes. As patients age, the severity of cervical degeneration tends to increase, leading to further degeneration of the intervertebral discs and structural imbalances in the cervical spine (23, 24). This aligns with studies showing that patients >60 years exhibit slower postoperative neurological recovery, with JOA score improvements lagging behind younger cohorts by approximately 8.3% (elderly recovery rate: 42.8% ± 28.5% vs. younger: 51.1% ± 32.2%, *p* < 0.05) (25). The reduced recovery in elderly patients correlates with lower preoperative JOA scores and age-related physiological limitations, though surgical intervention remains beneficial across all age groups (25, 26). These changes can exacerbate nerve root compression, and elderly patients often have weakened bone metabolism and repair functions, which can impede postoperative rehabilitation (27).

Bone mineral density was also found to be a key factor. The poor bone mineral density observed in the observation group is often associated with osteoporosis. Our findings corroborate recent evidence that low BMD, particularly T-scores < −2.0, significantly increases the risk of pseudoarthrosis and other mechanical complications after fusion surgery, as reduced BMD directly compromises implant stability and fusion success (28, 29). Specifically, osteoporotic patients (T-score ≤ −2.5) exhibit higher rates of pseudarthrosis and unplanned revisions due to weakened bone microstructure impairing graft integration, while low BMD also elevates risks of cage subsidence, screw loosening, and vertebral collapse (28, 29). Low bone density weakens the stability of the fusion segment, affecting the stability of the implant and hindering the fusion process. This significantly impacts the success of the surgery and its therapeutic efficacy.

Based on our findings, the severity of the patient's initial condition was another critical factor. The lower preoperative JOA scores and smaller D-values in the observation group suggested that their baseline condition was more severe. The 35% poor outcome rate (28/80) in our cohort aligns with historical controls reporting 30%–40% suboptimal results in patients with advanced degeneration (30). Severe initial conditions may result in significant neurological impairment. Although surgery can alleviate compression, the recovery of neurological function may take longer, emphasizing the importance of postoperative rehabilitation (31–33).

Postoperative complications were also found to play a significant role. Almost half of the patients in the observation group experienced postoperative complications. Notably, our pseudoarthrosis rate of 35.7% in the observation group exceeds rates reported with modern stand-alone or zero-profile implants (10%–20%) (34), potentially reflecting the older design of the BAK/C cage and the higher-risk profile of this patient subgroup. These considerations are critical as alternative motion-preserving strategies, such as cervical disc replacement (CDR), present different risk-benefit profiles, including lower pseudoarthrosis risk but potential for heterotopic ossification (35). In multi-level disease, the debate between fusion, arthroplasty, and hybrid surgery further underscores that surgical choices must be highly individualized to the patient's anatomy, bone quality, and functional goals (36).

It is also important to acknowledge the potential for selection bias inherent in this study's retrospective design. The allocation of patients into “favorable” and “poor” outcome groups was determined post-hoc, based on their 3-year results. Consequently, the observed significant differences in baseline characteristics, such as age and preoperative JOA scores, are expected findings that may reflect the natural history of the disease in these subgroups. While our multivariate logistic regression was employed to statistically adjust for these baseline differences and identify independent predictors, this observational design cannot establish causality. Future prospective studies are warranted to corroborate these risk factors in a more controlled setting.

## Study limitations

Our findings should be interpreted in light of several limitations. First, the retrospective design introduces potential selection bias and unmeasured confounders. While we standardized the surgical team and primary implant, variations in operative time, blood loss, and the selective use of anterior plating in multi-level cases were not included in the main regression model and could have influenced outcomes. Second, the sample size (*n* = 80), while providing adequate power for the primary analysis, was insufficient for internal validation by splitting the data into training and testing sets. The absence of an external validation cohort means our findings require confirmation in other patient populations. Third, the 3-year follow-up precludes assessment of very late complications like adjacent segment degeneration. Finally, pseudoarthrosis was diagnosed radiographically with mandatory CT confirmation for suspected cases, but the absence of routine CT for all patients may underestimate its true prevalence (37).

## Clinical implications

The identified risk factors (age, bone density) highlight actionable targets for preoperative optimization. For elderly patients, preoperative bone density screening and anti-osteoporotic therapy could enhance fusion success (38). In severe degenerative cases, hybrid techniques combining ACDF with dynamic stabilization may mitigate adjacent-level risks (39). Emerging technologies like 3D-printed titanium cages show promise in improving fusion rates in osteoporotic patients (40), warranting further comparative studies.

## Conclusion

This study demonstrates that age ≥55 years and reduced bone mineral density (T-score < −1.5) are critical determinants of suboptimal outcomes following ACDF with BAK/C interbody fusion. The high pseudoarthrosis rate (35.7%) in at-risk patients underscores the necessity for preoperative bone density optimization and adoption of enhanced fusion technologies. These findings emphasize a precision medicine approach to surgical candidate selection and perioperative management in cervical degenerative disease.

## Data Availability

The original contributions presented in the study are included in the article/Supplementary Material, further inquiries can be directed to the corresponding author.
